# Differences Between Traumatic and Degenerative Medial Meniscus Posterior Root Tears: A Systematic Review

**DOI:** 10.1177/03635465241237254

**Published:** 2024-04-10

**Authors:** Kristine Mundal, Andrew G. Geeslin, Eirik Solheim, Eivind Inderhaug

**Affiliations:** * Department of Clinical Medicine, University of Bergen, Bergen, Norway; † Haukeland University Hospital, Bergen, Norway; ‡ Larner College of Medicine, University of Vermont, Burlington, Vermont, USA; § Aleris Nesttun Bergen, Bergen, Norway; Investigation performed at the Department of Clinical Medicine, University of Bergen, Bergen, Norway

**Keywords:** medial meniscus posterior root tear, meniscus root tear, traumatic meniscus root tear

## Abstract

**Background:**

Intact meniscus roots are a prerequisite for normal meniscal function, including even distribution of compressive forces across the knee joint. An injury to the root disrupts the hoop strength of the meniscus and may lead to its extrusion and the development of osteoarthritis. A medial meniscus posterior root tear (MMPRT) is often thought to have a primary degenerative pathogenesis. However, there is mention of some cases of MMPRTs where the patients have a solely traumatic injury to a previously healthy meniscus.

**Purpose:**

To describe a subpopulation of patients with traumatic MMPRT.

**Study Design:**

Systematic review; Level of evidence, 5.

**Methods:**

The Web of Science database (www.webofscience.com) was queried using the Medical Subject Headings term “medial root tear.” Articles were reviewed, and those evaluated for MMPRTs in a degenerative meniscus were excluded. A total of 25 articles describing cases of acute traumatic causes were included in this study. For these articles, the patient characteristics, injury mechanisms, and concomitant injuries evaluated were recorded and pooled.

**Results:**

The search revealed 660 articles, and 25 were selected for inclusion. A total of 113 patients with a traumatic MMPRT were identified and included in this review. The study population had a mean age of 27.1 years and a high share of men (64%). Also, this review displays how most patients with traumatic MMPRTs also suffer concomitant injuries (68%).

**Conclusion:**

The findings in this review support our hypothesis that there is a unique subgroup with acute traumatic MMPRTs that have unique patient characteristics, injury mechanisms, and combined injuries, compared with previously published reviews on MMPRTs.

The menisci are critically important in maintaining a functional and healthy knee because of their role in force distribution and joint stability.^2,9,38^ Their attachment to the tibial plateau at the meniscus roots supports the conversion of the axial load from the femur into circumferential hoop stresses and results in an even distribution of forces at the articular cartilage.^2,6,42^ Loss of the root attachment leads to meniscal extrusion and increased peak cartilage contact pressure because of the reduced contact area.^10,38,39,52^ The link between meniscal dysfunction and osteoarthritis development has been well-established, especially in the setting of meniscus root tears.^5,11,16,34,35,38^

A meniscus root tear is defined as either an avulsion at the bony attachment site on the tibia or a complete radial tear within 1 cm from the insertion site.^36,38^ The lateral meniscus posterior root tears are more often found as a concomitant injury to an anterior cruciate ligament (ACL) tear than the medial meniscus posterior root tears (MMPRTs).^2,38,46^ The MMPRT has historically been characterized as a precursor to degenerative joint disease.^22,32,41,46^ There is a common agreement on the need to repair lateral meniscus posterior root tears.^39^ However, MMPRTs are often characterized as occurring secondary to knee degeneration rather than trauma, and there is debate on the efficacy of repair.^40^ Factors commonly associated with degenerative MMPRT are obesity, female sex, older age, and varus alignment.^3,14,15,21,64^ Although much of the scientific literature is focused on degenerative MMPRT,^1,4,32,53,54^ acute traumatic MMPRTs may also occur. Acute traumatic MMPRTs are likely better operative candidates because of tissue quality and the absence of degenerative changes secondary to the tear, and this group should be distinguished from patients with a degenerative origin.^1,54^ However, the studies reporting on patients with MMPRT are often small and heterogeneous, and the knowledge on this subgroup of traumatic root tears is therefore fragmented and requires investigation through a focused systematic review. The aim of this study was to systematically review the literature to identify patients with traumatic MMPRT. We hypothesized that there would be a subgroup of patients with MMPRT with a traumatic origin rather than a degenerative pathogenesis and that these patients would have different demographic characteristics and injury mechanisms when compared with the general population that sustains MMPRT.

## Methods

Using the PRISMA (Preferred Reporting Items for Systematic Reviews and Meta-Analyses) guidelines, a systematic literature review was performed on December 28, 2022, using “medial root tear” as a Medical Subject Headings term in the Web of Science database (www.webofscience.com). Only publications with either an English or a German abstract published after January 1, 2002, were considered eligible for inclusion.

To identify articles presenting MMPRT cases with an acute traumatic origin, the first author (K.M.) reviewed all titles, abstracts, methods, and patient characteristics. An acute traumatic origin was defined as a specific traumatic event with a detailed injury mechanism or with a severity of injuries, suggesting that the injury event must be acute traumatic. Thereafter, the full-text publications were reviewed to finalize the inclusion and extract data. Reference lists of selected articles were reviewed to search for additional relevant publications. Articles without patient-level data or with a clear degenerative origin of the MMPRT were excluded. Editorial comments, technical notes only focusing on surgical methods, biomechanical studies on cadaveric specimens and animals, and other reviews and meta-analyses were also excluded. Corresponding authors were contacted by email to request missing data or clarify whether or not patients’ injuries were caused by trauma. We excluded patients with Kellgren-Lawrence grades of >1 or Outerbridge classification grades of >2 to focus on acute traumatic injuries. To limit the inclusion of patients with a likely degenerative cause of their meniscal tear in cases where there was doubt about whether articles should be included, a discussion among the first author and 2 of the senior authors (A.G.G. and E.I.) was held to reach a consensus.

## Results

**Figure 1.**
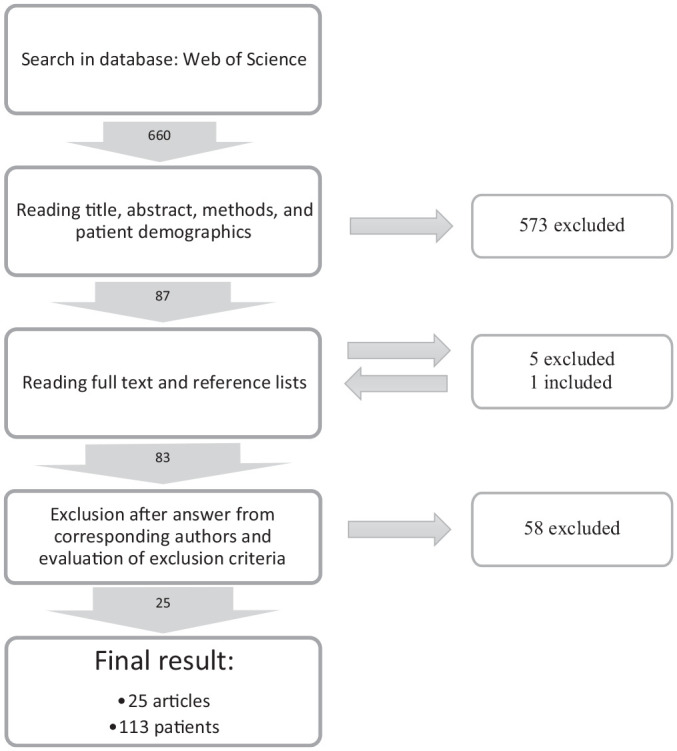
A flowchart for the inclusion of data for review.

The search yielded a total of 660 articles. After screening the titles, abstracts, methods, and patient characteristics, 87 articles were included for full-text review (Figure 1). Out of 60 corresponding authors contacted, 28 replied and helped clarify the inclusion/exclusion criteria by adding further patient-level data. After full-text review, obtaining supplemental injury information from the corresponding authors (if necessary), and author discussion, 25 articles were selected for the final inclusion (Figure 1).

In total, 113 patients, with a mean age of 27.1 years (range, 12-61 years [SD, 11.2]), were pooled for analyses. Overall, sex was reported for 93 patients—21 women and 72 men (Table 1).

**Table 1:** Patient Characteristics Across the Included Studies^*a*^

Paper No.^*b*^	Authors	Study Design	Year of Publication	No. of Patients	Sex^*c*^	Age,^*d*^ Years
1^8^	Engelsohn et al	Case report	2007	2	1M + 1F	19.5
2^55^	Ra et al	Case series	2015	7	6M + 1F	28.9
3^29^	Kidron and Thein	Case series	2002	11	10M + 1F	27.4
4^19^	Hiranaka et al	Case report	2019	1	1M	34
5^24^	Jones et al	Review with case reports	2010	5	1M + 4F	45.2
6^30^	Kim et al	Case series	2010	10	10M	30.6
7^43^	Lee et al	Case report	2009	1	1F	54
8^56^	Seil et al	Case report	2011	1	1M	21
9^61^	Wilson and Johnson	Case report	2011	2	1M + 1F	16.5
10^9^	Feucht et al	Case report	2014	1	1M	17
11^31^	Koenig et al	Review with case report	2009	1	1M	15
12^62^	Wilson et al	Cohort study	2018	12	10M + 2F	15.3
13^22^	Iversen and Krogsgaard	Case report	2014	2	1M + 1F	12.5
14^63^	Xue et al	Surgical note with case reports	2018	3	3M	43
15^59^	Sonnery-Cottet et al	Case report	2014	2	2M	13
16^60^	Tenfelde et al	Case report	2022	1	1F	20
17^45^	Matava and Kim	Case report	2011	1	1M	12
18^47^	Naraghi and White	Review with case report	2016	1	1M	39
19^44^	Marzo	Review with case report	2009	1	1M	43
20^57^	Sharif et al	Review with case report	2020	1	1F	26
21^7^	Dzidzishvili et al	Case-control study	2021	18	16M + 2F	28
22^32^	Kosy et al	Case series	2018	20		
23^28^	Karpinski et al	Cohort study	2023	6	4M + 2F	36.3
24^58^	Shieh et al	Cross-sectional study	2013	2	2F	15
25^23^	Jones et al	Review with case report	2006	1	1F	15

a F, female; M, male.

b The superscripted numbers refer to numbers in the reference list.

c The number of male and female patients.

d Age is presented in means.

Detailed reports on injuries other than the meniscal tear were available for 106 patients (94%) (Table 2 and Appendix Table A1, available in the online version of this article). A total of 77 patients had such concomitant injuries (68%), while 29 patients had an isolated MMPRT (26%). Of patients with concomitant injuries, 74 (66%) had injuries that involved either the ACL (n = 32 patients), the posterior cruciate ligament (PCL) (n = 10 patients), or both (n = 32 patients) (Appendix Table A1, available online). Using the Schenck Classification of Knee Dislocations,^10^ (KD I-V), the most common group was the KD I, which applied in 42 patients (37%), followed by the KD III (n = 14 patients) and the KD V (n = 10 patients). The KD III involves both the ACL, the PCL, and 1 of the collateral ligaments. The KD V is defined as a multiligament injury combined with a periarticular fracture. Some of the patients had other injuries (n = 22 patients)—such as fractures, chondral damage, and concomitant lateral root tear (Table 2). These pathologies occurred with the MMPRT, in combination with one another, or in combination with tears of the cruciate ligaments.

**Table 2:** Distribution of the Schenck Classification and Other Concomitant Injuries Across Different Types of Traumatic Events^*a*^

	Injury Mechanism					
Concomitant Injuries	High Energy/Trauma Injury	Sports Trauma	Fall Injury	Various Trauma	Unknown Trauma	Total
KD I	4	27	4	3	4	42
KD II	0	0	0	0	1	1
KD III	5	5	1	0	3	14
KD IV	0	0	0	0	7	7
KD V	1	1	0	0	8	10
Other concomitant injuries^*b*^	0	9	1	3	0	13
No concomitant injuries	0	18	3	8	0	29
Unknown	0	5	0	1	1	7

a KD, Schenck Classification of Knee Dislocations; LMPRT, Lateral meniscus posterior root tear.

b Other concomitant injuries included injuries such as fractures, LMPRT, and chondral lesions.

The injury mechanism for each patient was reviewed to allow grouping into categories depending on the injury event—including traffic accident, sporting trauma, fall, various known trauma, and unknown trauma (Table 2). The mechanism was a traffic accident for 10 patients, all of whom had a concomitant ligament injury—including the ACL, the PCL, or both. Sporting injuries made up the largest group of patients (n = 59). The injuries were sustained during several sporting activities—such as football, soccer, basketball, and so forth. These patients had a binary distribution, as they either suffered from an isolated root tear or a tear combined with a ligament injury. The highest number of isolated root tears was found in this group. In patients with a fall as the documented mechanism of injury (n = 8), there were a variety of concomitant injuries—including PCL tears, ACL tears, and chondral lesions. The group that denoted “various known trauma” included 13 patients and had the highest share of isolated tears (62%)—with only a few patients with single ligament tears and none with a multiligament injury.

In 23 out of the 113 patients, the injury mechanism was not distinguishable at a patient level. Of these injuries, 20 were pooled as traffic accidents or sports injuries in the original publication and were denoted “other injuries” for the analyses. All the patients in this group suffered from ligament tears; most had a multiligament tear (n = 19 patients). None of the patients in this group had an isolated MMPRT.

## Discussion

Traumatic MMPRTs have not been reviewed since Feucht et al^9^ evaluated this group in 2014. The present analyses found a relatively young age in the present pooled patient cohort, with a mean age of 27.1 years. This included a significant number of patients <30 years old (62%) and patients <40 years old (72%) at the time of injury. The oldest patient, included in the study by Jones et al,^24^ was 61 years old at the time of injury. This article also included a 52-year-old patient. Another paper, by Xue et al,^63^ included a patient aged 59 years. Two other studies included patients aged 54 years (Lee et al^43^ and Karpinski et al^28^). In contrast to the young age of patients in this review, several reviews on MMPRT have reported^16,21,33^ that mean patient age ranged from 51.4 to 58.2 years. Also, the presence of degenerative cartilage—classified by radiographs or arthroscopic inspection—was low across the included studies. Studies by Kamimura et al,^26,27^ Kamatsuki et al,^25^ and Okazaki et al^48^ showed that 50% to 69% of patients had a Kellgren-Lawrence grade of >1 at the time of surgery. This indicates some level of osteoarthritis/chondral wear. The younger age and lower incidence of chondral wear suggest that traumatic MMPRT seems to be a different entity from those with a dominant degenerative path.

Our study population also showed a different sex distribution, with 64% being men and 19% being women, whereas most other reports on MMPRT predominantly involve women. The study by Hwang et al^21^ included 94% women. Okazaki et al^48-51^ reported between 80% and 85% women in their MMPRT groups across several articles. Studies from Hiranaka et al^17,18,20^ reported that between 69% and 93% of their study populations were women. In a report by Krych et al,^33^ in which the medial and lateral root tears were compared, 56% of women were reported to be in the MMPRT group. In that study, it was noted that the lateral meniscus posterior root tears group is younger and usually has concomitant ACL tears, suggesting a traumatic origin—whereas the MMPRT group is older and has more concomitant degenerative changes. The impression from reading the literature is that a standard MMPRT patient is a middle-aged woman—while the present review shows that this might be completely different for traumatic MMPRTs. This finding aligns with the hypotheses of LaPrade et al^37^ in a recent editorial commentary.

When considering injury mechanisms, the most frequently reported cause of tear across the cohort was sports-related trauma, followed by unknown trauma and various known traumas such as those during jumping, bicycling, fitnesss, and so forth. The level of detail in the included studies also allowed analyses showing that most patients had a concomitant injury (68%). These were most frequently ligamentous injuries, fractures, chondral damages, or some combination of these. Ligamentous injuries made up the biggest group of concomitant injuries and were further categorized using the KD.^13^ The relatively high degree of KD III to V injuries, involving multiple ligaments and fractures, shows how high energy has likely been involved in most of the injury settings. This contrasts with a degenerative tear that can debut spontaneously or during light activities such as walking, turning, or light running^10,22^—often referred to as microtrauma.

Another sign of high forces acting on the knee at injury is the low level of isolated meniscal tears (26%). This is not surprising, however, as the medial meniscus acts as a secondary stabilizer for the anterior translation of the knee^12,40^ and likely also plays a role in restraining anteromedial/posteromedial rotation. For a healthy medial meniscus to sustain forces large enough to cause a lesion in its posterior root, it almost seems necessary that other structures tear in that same sequence of injuries. Although there is very little evidence linking specific trauma mechanisms to meniscal tear types, one could speculate—based on basic biomechanical knowledge—whether a common injury mechanism for an MMPRT tear combines knee torque, valgus load, flexion, or hyperextension.^12^ A few included articles have described injury mechanisms in detail and found such a combination of forces. Overall, it was difficult to identify any common injury patterns based on the included studies, as several other injury mechanisms were also observed.

There are some limitations to this review. The individual age and sex of patients were not available because of incomplete reporting in the index studies and unavailability upon contacting study authors. Furthermore, we did not have complete details about patients’ grades of osteoarthritis—defined as the Kellgren-Lawrence grade, the Outerbridge classification, any other classification, or the body mass index for all patients. Although we inquired about osteoarthritis classification from all corresponding authors, only a minority of patients had such data at final analyses. Given the relatively young age and known traumatic event of the collapsed cohort, authors may not have found it necessary to seek and include this information. More such details could have further supported or refuted our hypothesis that degenerative and traumatic MMPRTs have different demographic and injury-related characteristics.

## Conclusion

The findings in this review support our hypothesis that there would be a unique subgroup with acute traumatic MMPRTs that would have unique patient characteristics, injury mechanisms, and combined injuries compared with previously published reviews on MMPRTs.

## Supplemental Material

sj-pdf-1-ajs-10.1177_03635465241237254 – Supplemental material for Differences Between Traumatic and Degenerative Medial Meniscus Posterior Root Tears: A Systematic Review Supplemental material, sj-pdf-1-ajs-10.1177_03635465241237254 for Differences Between Traumatic and Degenerative Medial Meniscus Posterior Root Tears: A Systematic Review by Kristine Mundal, Andrew G. Geeslin, Eirik Solheim and Eivind Inderhaug in The American Journal of Sports Medicine
