# Supplementary material for: Differences Between Traumatic and Degenerative Medial Meniscus Posterior Root Tears: A Systematic Review
Source: Am J Sports Med. 2024 Apr 10;53(1):228–33. doi: 10.1177/03635465241237254 (PMC11689786; doi:10.1177/03635465241237254)
Supplement: sj-pdf-1-ajs-10.1177_03635465241237254 – Supplemental material for Differences Between Traumatic and Degenerative Medial Meniscus Posterior Root Tears: A Systematic Review [file sj-pdf-1-ajs-10.1177_03635465241237254.pdf]

Are there any differences between traumatic and degenerative medial meniscal posterior root tears?

Appendix

Table A1: Injury mechanism and concomitant injuries

Paper number*	Authors	Number of patients	Injury mechanism	Concomitant injuries**
1[8]	Engelsohn E. *et al.*	Total number of patients: 2		
		1	Hit by car	PCL tear, ACL tear, MCLt tear, reversed Segond fracture, patellar dislocation
		1	Soccer, another player stepped on his knee	PCL tear, ACL tear, FCL, BFT tear, tibial plateu fracture
2[55]	Ra H.J. *et al.*	Total number of patients: 7	Hyperextension with valgus trauma	7 MCL tear + posteromedial capsule injury; 3 ACL tears, 1 PCL tear, 1 pPCL tear, 2 PCL+ACL tears
		2	Snowboard	
		1	Motorcycle	
		1	Pedestrian accident	
		1	Ice hockey	
		1	Soccer	
		1	Taekwondo	
3[29]	Kidron A. *et al.*	Total number of patients: 11	Acute flexion injury	1 pACL tear, 1 chondral lesion
		3	Soccer	
		1	Basketball	
		1	Volleyball	
		1	Aerobics	
		1	Handball	
		1	Gymnastics	
		2	Soldier	
		1	Judo	
4[19]	Hiranaka T. *et al.*	Total number of patients: 1	Soccer	ACL tear bilateral
5[24]	Jones C. *et al.*	Total number of patients: 5		

		4	Twisting injury	None
		1	Former ACL-injury	ACL - graft failure
6[30]	Kim Y.J. *et al.*	Total number of patients: 10		
		1	Fall down	ACL tear
		1	Soccer	PCL tear, PLC
		1	Soccer	ACL tear
		1	Motorcycle	PCL tear, PLC, coronary ligament tear
		1	Motorcycle	ACL tear, PCL tear, PMC, MCL tear
		1	Pedestrian accident	ACL, PCL, PLC
		1	Fall down	ACL tear,, PCL tear, PLC, MCL tear
		1	Ski	ACL tear, ALC
		1	Fall down	ACL tear
		1	Motorcycle	PCL tear, MCL tear, coronary ligament tear
7[43]	Lee J.H. *Et al*	1	Fall from height	LMPRT, ACL tear
8[56]	Seil R. *Et al*	1	Football injury	cartilage lesion - medial femoral condyle
9[61]	Wilson B.F. *Et al*	Total number of patients: 2		
		1	Soccer	ACL tear, pPCL tear, MCL tear
		1	Traffic accident, ejected from motor vehicle.	ACL tear, PCL tear, MCL tear
10[9]	Feucht M.J. *Et al*	1	Jump, twisted kne when landing	ACL tear, LMPRT
11[31]	Koenig J.H. *Et al*	1	Hit by motor vehicle while cycling.	ACL tear, PCL tear, MCL tear
12[62]	Wilson P.L. *Et al*	Total number of patients: 12	Contact, non-contact or high-velocity trauma	
		1	Soccer	ACL tear, LMPRT, chondral lesions
		1	Football	ACL tear, LMPRT, chondral lesions
		1	Soccer	ACL tear, LMPRT, chondral lesions
		1	Bicycle accident	tibial spine fracture, chondral lesions
		1	Football	None
		1	Fall on playground	None
		1	Basketball	ACL tear

		1	Football	Multi-ligament injury, chondral lesions
		1	Motorcross	Multi-ligament injury
		1	Soccer	ACL
		1	Football	None
		1	Football	Multi-ligament injury
13[22]	Iversen J.V. *Et al*	Total number of patients: 2		
		1	Ski accident, distortional trauma	None
		1	Trampoline accident, distortional trauma	None
14[63]	Xue H.H. *Et al*	Total number of patients: 3		3 PCL tears
15[59]	Sonnery-Cottet B. *Et al*	Total number of patients: 2		
		1	Tortional trauma, fall when roller-skating	None
		1	Tortional trauma, playing football	None
16[60]	Tenfelde O. *Et al*	1	Jump from 2.5 meter. Hyperflexion varus trauma	None
17[45]	Mataya M.J. *Et al*	1	Ski accident, tortional trauma with valgus.	None
18[47]	Naraghi A.M. *Et al*	1	Hyperextension with valgus trauma	pPCL tear, POL tear
19[44]	Marzo J.M. Et al	1	Roof collapse, fall.	ACL tear
20[57]	Sharif B. *Et al*	1	Football injury	Unknown
21[7]	Dzidzishvili L. *Et al*	Total number of patients: 18	Sports injuries, soccer etc.	11 ACL-tears
22[32]	Kosy J.D. *et al*	Total number of patients: 20	High energy trauma (traffic accident etc.) Low energy trauma (sports, exercise etc.)	20 multiple ligament injuries
23[28]	Karpinski K. *et al.*	Total number of patients: 6		
		1	Soccer	Unknown
		1	Handball	Unknown
		1	Climbing stairs	Unknown

		1	Wakeboard	Unknown
		1	Volleyball	Unknown
		1	Horse riding	Unknown
24[58]	Shieh A. *et al.*	Total number of patients: 2		
		1	Wrestling	ACL tear
		1	Sports activities	ACL tear
25[23]	Jones O.A. *et al.*	1	Soccer	PCL tear

Superscripted numbers refer to numbers in the reference list, *****Abbreviations: ACL*** *– Anterior Cruciate ligament,* ***pACL*** *– partial ACL tear,* ***PCL*** *– Posterior Cruciate ligament,* ***pPCL*** *– partial PCL tear,* ***MCL*** *– Medial collateral ligament*, ***FCL*** *– fibular collateral ligament* ***PLC*** *– posterolateral corner,* ***PMC*** *– posteromedial corner,* ***BFT –*** *biceps femoris tendon,* ***LMPRT*** *– lateral meniscal posterior root tear,* ***POL*** *– posterior oblique ligament.*
